# Romantic Attachment and Difficulties in Emotion Regulation on Dyadic Adjustment: A Comprehensive Literature Review

**DOI:** 10.3389/fpsyg.2021.723823

**Published:** 2021-12-13

**Authors:** Marisalva Fávero, Lúcia Lemos, Diana Moreira, Filipe Nunes Ribeiro, Valéria Sousa-Gomes

**Affiliations:** ^1^Department of Social and Behavioral Sciences, University of Maia (ISMAI), Maia, Portugal; ^2^Unit I&D of the Justice and Governance Research Center of the Law School, University of Minho, Braga, Portugal; ^3^University Fernando Pessoa, Porto, Portugal; ^4^Laboratory of Neuropsychophysiology, Faculty of Psychology and Educational Sciences, University of Porto, Porto, Portugal; ^5^Institute of Psychology and Neuropsychology of Porto – IPNP Health, Porto, Portugal; ^6^Centro de Solidariedade de Braga/Projecto Homem, Braga, Portugal

**Keywords:** romantic relationships, romantic attachment, difficulties in emotional regulation, dyadic adjustment, literature review

## Abstract

In romantic relationships, individual differences are determinant factors for relational quality. Specifically, romantic attachment (RA) and difficulties in emotional regulation influence each other and may have predictive potential for the perceived dyadic adjustment (DA) level. This paper aims to identify the developmental parallel between behavioral patterns built since childhood and the construction of the emotional regulation skills that characterize them. Our analysis was based on the attachment theory and the concepts of romantic relationship and DA. In this way, we sought to further the understanding of relationship dynamics, beyond the usual focus on a single element and on associative relationships, and by exploring other effects among the different dimensions of relational functioning. In particular, we explored the predictive ability of emotional regulation patterns (more flexible individual characteristics) in discriminating between RA styles (more perennial influences), and their impact on the quality of romantic relationships, in the anticipation of dyadic adjustment variations.

## Introduction

A romantic relationship is characterized by feelings of trust, as well as close and intense interaction (Regan et al., 1998; Muise et al., 2018), generally including an intimate sexual dimension as a distinctive element (Meltzer et al., 2017; Maxwell and McNulty, 2019). It is defined by having its own identity, specific rules, and a trajectory (Finkel et al., 2017; Porreca, 2019) which is often unpredictable (Eastwick et al., 2018).

A trusting relationship is expected to have a certain degree of predictability, with expectations being created about fulfilling each partner’s needs, and creating a balance between a necessary and sufficient degree of relational dependence and the autonomy of each partner (Coimbra de Matos, 2017; Finkel et al., 2017; Overall and Cross, 2019), contributing to stability, commitment, and satisfaction (Spielmann et al., 2013; Wang, 2019, Unpublished). However, these expectations can also generate insecurity when faced with doubts about alternatives to satisfy needs in the absence of the relationship (Attridge et al., 1998). Still, despite the associated risks, most people feel motivated and desire to find, engage in, and maintain a romantic relationship (Bookwala, 2015; Joel et al., 2019).

According to attachment theory, one of the most frequently applied and influential in the study of romantic relationships (Finkel et al., 2017), trust is a differentiating factor concerning the representations of relationship that each partner brings into the couple. This dimension underlies predispositions and expectations about the other and about the future of the relationship (Campbell and Stanton, 2019), in addition to playing a protective role against relational problems (Shallcross and Simpson, 2012).

On the one hand, regardless of their degree of commitment or type, relationships tend to be a source of well-being (Farero et al., 2019; Waddell et al., 2019), and are associated with better physical and mental health (Braithwaite and Holt-Lunstad, 2017; Slatcher and Selcuk, 2017; Wang et al., 2017), as well as to reports of greater happiness (Kawamichi et al., 2016) and satisfaction with life (Gustavson et al., 2016). The absence of a relationship, on the other hand, seems to be associated with the perception of greater loneliness and lower social support, with negative indirect effects on life satisfaction (Adamczyk and Segrin, 2015). In addition, relational disruptions cause multiple consequences and substantial losses in different areas of life, negatively impacting physical and mental health (Fox and Tokunaga, 2015; Fincham et al., 2018).

Many individuals aspire to have a stable and lasting romantic relationship, which has significant implications for quality of life, from the point of view of the individual, the couple, or even society itself, making it an important topic of study (Spanier, 1976). Thus, understanding which factors are involved in relationship functioning, and how they are articulated has been an important focus of researchers (Bookwala, 2015; Bertoni et al., 2020).

This paper stems from a description of the concepts of romantic relationship and dyadic adjustment (DA), which is a comprehensive indicator of the perception of relational quality. We aim to expand our focus beyond the assessment of satisfaction, which is a more frequent target in this research field.

Difficulties in adapting to a shared life (Costa et al., 2016; Cho et al., 2020) or the breaking of trust (Wang, 2019, Unpublished) often generate marital conflicts and/or emotional distress. These result from a variety of causes linked to individual characteristics, communication, the couple’s routines, and surrounding contexts (Costa et al., 2016; Doss and Rhoades, 2017), causing damage to the couple’s bond (Warach and Josephs, 2019). Attempts to resolve these difficulties prove to be more effective in couples with better relational skills, who are able to project and reflect about the future of the relationship, with mutual consideration, openness and security, ability to communicate and regulate emotions (Costa et al., 2016), as well as share tasks and responsibilities (Costa et al., 2016; Weiser and Weigel, 2016; Davila et al., 2017).

When targeting relational difficulties or conflicts, the benefits and effectiveness of therapeutic interventions (Snyder and Halford, 2012; Davila et al., 2017; Wiebe et al., 2017) are still insufficient to mitigate negative consequences, namely regarding dissolution of relationships, including divorce (Røsand et al., 2014; Pordata, 2020). This emphasizes the relevance of research in this field, to guide practice at the individual, family, and/or couple level, as well as help identify and characterize mechanisms and models that govern or allow to predict how relationships develop, evolve (Consoli et al., 2018; Farero et al., 2019), and are reconstructed (Limeira and Féres-Carneiro, 2019).

Despite some theoretical and conceptual dispersion, the findings from research on romantic relationships tend to be coherent (Rogge et al., 2017). Generally, they focus on concepts related to relational quality (Fowers et al., 2016), frequently quantified by DA – a concept of difficult interpretation – which describes the dynamic process of the romantic partnership interaction and relation (Gomez and Leal, 2008; Rosado and Wagner, 2015; Rosado et al., 2016).

### Dyadic Adjustment

The DA, or coordination between the members of a romantic couple, emerges at the beginning, and continues throughout the relationship, in a process through which partners adapt to each other and to their roles and responsibilities in the relationship (Kendrick and Drentea, 2016). The adequacy achieved in the relationship can potentially discriminate its degree of viability (Montesino et al., 2013; Kendrick and Drentea, 2016). As a measure of adjustment, DA implies the creation and maintenance of a flexible, but reliable, agreement about what the relationship needs in order to function, taking into consideration mutual discovery without neglecting respect for individual development (Rao, 2017).

A better DA is observed in a greater tendency to function as a team, which requires cognitive, emotional, and social maturity, as well as altruism on both sides to accommodate the other’s satisfaction over an exclusive focus on the individual (Kendrick and Drentea, 2016; Rani et al., 2017; Rao, 2017). Therefore, it requires a commitment toward the partner and the relationship that generates cohesion, and a search for agreement on shared issues that allows for good communication, consensual decisions, and effective conflict management (Kendrick and Drentea, 2016).

Research often uses DA as a result variable to assess how the quality of romantic relationships can be impacted by factors of an individual, contextual, and relational nature (Rosado et al., 2016; Cho et al., 2020). For Spanier (1976), this individual subjective perception is obtained from three components: (1) satisfaction or happiness in the relationship; (2) the couple’s cohesion regarding shared activities; and (3) consensus on relationship-specific issues, including the expression of affection. The data gathered allows for a qualitative differentiation between relationships (Spanier, 1976; Farero et al., 2019), in a longitudinal dynamic perspective, as evolving processes (Spanier, 1976; Scorsolini-Comin and dos Santos, 2012).

Thus, the perceived relational quality is inseparable from how close relationships are experienced, including romantic ones. These experiences, the satisfaction they provide, and the ease or difficulty with which they are managed, vary substantially according to individual attachment characteristics that predispose people to adopt a certain set of behaviors in relationships (Fraley and Hudson, 2017; Fraley and Roisman, 2019), which dictate hopes and fears in selecting, constructing, experiencing, and maintaining relationships (Bowlby, 1969, 1982; Ainsworth, 1979; Ainsworth et al., 2015; Fraley and Hudson, 2017; Fraley, 2019; Fraley and Roisman, 2019).

### Attachment and Romantic Relationships

The individual differences, evidenced in relational patterns throughout life (Fraley and Hudson, 2017; Simpson and Rholes, 2017; Theisen et al., 2018), can be explained using mental models that, according to attachment theory, are internalized within the first social interactions with primary caregivers. These experiences create representations about the self and others, which become the guiding framework for expectations and responses to future relationships (Bowlby, 1969, 1982; Ainsworth, 1979; Sroufe, 2016; Simpson and Rholes, 2017; Eppel, 2018).

Attachment can be understood as an innate motivational system, which has evolved to guide behavior toward survival and adaptation (Fraley and Hudson, 2017), by promoting a lasting emotional connection (Fraley, 2019) as a safe base and haven (Hazan and Shaver, 1994; Ainsworth et al., 2015; Gillath et al., 2016; Simpson and Rholes, 2017). Ainsworth et al. (2015) differentiated the internalized models according to the conduct of the primary attachment figure. When this figure is responsive and available, the model is organized in a safe pattern, within experiences of care, comfort, protection, and alleviation of negative emotions, thus fostering confidence in the accessibility of help (Bowlby, 1969; Pascuzzo et al., 2013). This is not possible for insensitive or inconsistent caregiving attitudes, which enhance insecure patterns that will be anxious or avoidant (Fraley and Hudson, 2017; Simpson and Rholes, 2017; Umemura et al., 2018). Only the safe model allows independence from real proximity and transforms the relationship into a goal-corrected partnership, creating the conditions for a confident and autonomous exploration, including the establishment of secondary bonds (Hazan and Selcuk, 2015; Freeman and Simons, 2017; Mota, 2018).

These internal models permeate personality development, but not as a static phenomena (Pinquart et al., 2013; Groh et al., 2014; Theisen et al., 2018). If, on the one hand, they create a tendency for peer selection, on the other hand, they show susceptibility to the effects of socialization in new relationships (McConnell and Moss, 2011; Fraley and Hudson, 2017; Simpson and Rholes, 2017; Siegel et al., 2018; Fraley, 2019; Fraley and Roisman, 2019; Galaviz, 2019; Mikulincer and Shaver, 2019; Sutton, 2019), with this mutability being driven by the cumulative effect of continuous relational experiences (Van Ryzin et al., 2011; Simpson et al., 2015; Taylor et al., 2015; Gillath et al., 2016; Fraley and Hudson, 2017; Arriaga et al., 2018).

The acquisition of autonomy, in early adolescence (Theisen et al., 2018), tends to center the main bond on a romantic partnership (Freeman and Simons, 2017; Umemura et al., 2018; Fraley, 2019), involving a sexual aspect which definitely contrasts with early attachment (Ávila et al., 2011; Zeifman and Hazan, 2016). In addition, adult relationships will also differ due to their reciprocal, bidirectional, and voluntary nature, with parity of roles (Gillath et al., 2016) and susceptibility to dissolution (Simpson et al., 2015). Adult social empowerment and the accumulation of relational experiences, romantic or otherwise, enhance the complexity of developing bonds, particularly in romantic attachment (RA), which highlights the importance of its characterization (Hazan and Selcuk, 2015; Merrill, 2018; Fraley and Roisman, 2019).

In the process of building primary attachment, the experience of dyadic emotion regulation (ER) is a central aspect of human growth and development (Cole, 2014), because it establishes the foundations for affective regulation mechanisms (Cassidy, 1994; Viddal et al., 2017), which can be generalized to future interactions, including romantic relationships (Bowlby, 1969; Cassidy, 1994; Brenning and Braet, 2013; Pascuzzo et al., 2013; Sroufe, 2016).

### Emotion Regulation and the Quality of Romantic Relationships

Emotion regulation is a dynamic process that influences and is influenced by intra and interpersonal factors (Grandey and Melloy, 2017), neurologically materialized and inseparable from the quality of the primary caregiver relationship (Etkin et al., 2015; Eppel, 2018; Panksepp et al., 2019). The developed regulatory resources define the level of security or insecurity intrinsic to the internal relational models, differentiating them in terms of response to threatening stimuli (Brenning and Braet, 2013; Guzmán-González et al., 2016).

Insecure models manifest themselves between two extremes. One is anxiety/ambivalence, characterized by emotional hyperactivation and guided by fears of abandonment and lack of value, aiming to ensure and retain the availability of an unpredictable partnership (Brenning and Braet, 2013; Gillath et al., 2016; Winterheld, 2016). Another extreme is avoidance, which is characterized by hypoactivation/emotional deactivation, intending to inhibit negative emotions elicited by fear of rejection, in which discomfort with proximity and dependence promotes a refusal of support from the partner, who is seen as unreliable (Malik et al., 2015; Simpson and Rholes, 2017; Umemura et al., 2018; Mikulincer and Shaver, 2019).

In contrast, attachment security is characterized by low levels of anxiety and avoidance, and is considered a resilience resource when facing vulnerabilities and challenges, in which greater effectiveness in accepting, expressing, and using emotions improves the selection of regulatory strategies (Goodall, 2015; Mikulincer and Shaver, 2019) and lessens concerns with the responsiveness of others as sources of comfort and safety (Winterheld, 2016; Fraley and Hudson, 2017).

Hazan and Shaver (1987) were pioneers in expanding the theoretical attachment approach (Bowlby, 1969, 1982; Ainsworth, 1979) to romantic relationships. In this constantly evolving conceptualization (Siegel et al., 2018; Zeifman, 2019), romantic love is represented through flexible models, differentiated by the accumulation of relational experiences, in more or less safe patterns, that reverberate in future relationships (Godbout et al., 2017; Furman and Collibee, 2018; Girme et al., 2018; Fraley and Roisman, 2019).

Research on the impact of these differences has shown that secure patterns are characterized by partner support and acceptance, more effective emotional regulation and communication skills, the ability to identify and discuss negative experiences and resolve conflicts, with relationships being enjoyed with greater harmony, commitment, trust, and satisfaction (Gillath et al., 2016; Fraley, 2019). Insecure patterns, due to weak early development of coping resources, tend to welcome more negative perspectives and greater difficulties, concerns, and fears about intimacy (Mark et al., 2018; Birnbaum and Reis, 2019; Mikulincer and Shaver, 2019). The anxious/ambivalent extreme tends to be obsessive, emotional, and/or sexual, and characterized by jealousy or distrust, whereas individuals at the avoidant extreme exhibit discomfort with closeness and intimacy (Meyers and Landsberger, 2002; Simpson and Rholes, 2017).

Insecure attachments tend to have a negative impact on cognitive, emotional, and behavioral aspects of relational quality (Li and Chan, 2012), revealing a negative association with DA (Muraru and Turliuc, 2012). Couples in which both partners exhibit insecure RA tend to report lower DA, while the trend is reversed if both exhibit secure RA, with varying levels of DA being observed in couples with mixed attachment (Siegel et al., 2018). However, the mechanisms involved in the relationship between RA and DA remain relatively unknown (Martins et al., 2016). Thus, our objective is to contribute to their clarification.

### Attachment, Difficulties in Emotion Regulation, and Relationship Quality

Attachment remains, throughout the life cycle (Cole, 2014; Zimmermann and Thompson, 2014), inseparable from ER patterns (Hazan and Selcuk, 2015). This renders the attachment system to be considered an emotion regulating system (Gillath et al., 2016), in which regulatory skills work as an extension of the internal models that guide behavior within relationships (Malik et al., 2015; Gardner et al., 2019).

Therefore, when establishing and developing RA, the regulation of emotions by each partner will depend on the pattern defined in their primary emotional connection and under potential influence of successive later relationships, which will have repercussions on each new connection under construction. Regulatory patterns were identified as mediators in the development of representations of romantic relationships in adulthood, in their degree of insecurity, with unsecure standards contributing to the development of less confident bonds (Ávila, et al., 2011; Pascuzzo et al., 2013; Espeleta et al., 2017). They are responsible for effects on relational quality, suggesting tendencies for lower relational adjustment, as well as complex links between RA, ER, and relationship quality, which have yet to be examined (Brandão et al., 2019).

Emotions, whose function is to direct action toward goals and the fulfillment of needs (Elliott, 2012), inevitably affect the interaction of couples (Veloso et al., 2011). They influence satisfaction, quality, and success (Cohen et al., 2012; Bloch et al., 2014; Waldinger and Schulz, 2016), which can become impaired when verbal or bodily emotional manifestations (Barrett and Gross, 2001; Gross, 2001; Peña-Sarrionandia et al., 2015; Dworkin et al., 2018) are inadequate in type, moment, intensity, or quality (Gross, 2015).

The maladaptive relevance of difficulties in emotion regulation (DER; Fernandez et al., 2016) boosted an integrative and transversal view (Vaz, 2018), covering a wide spectrum of aspects. These include the ability for awareness, clear understanding and acceptance of emotions, impulsivity control, goal-oriented behavior in the presence of negative emotions, as well as the selection and contextualized application of ER strategies (Gratz and Roemer, 2004). When regulatory impairment affects emotional flexibility (Brenning and Braet, 2013), well-being and adaptation are compromised (Veloso et al., 2011), and the implications are not limited to the individual level (Gross, 2001, 2015; Bjureberg et al., 2016).

In this sense, the adjustment of the emotional experience is fundamental (Gross, 1998, 2001; Peña-Sarrionandia et al., 2015). In a dyadic relationship, this adjustment is a bidirectional co-regulatory process (Haase, 2014; Gross, 2015; Horn and Maercker, 2016), positively associated with the couple’s emotional satisfaction and stability (Butler and Randall, 2013; Rusu et al., 2019). Moreover, it has effects on the protection of relationships and the strengthening of emotional connections (Dworkin et al., 2018). In fact, emotional intelligence (EI), which is the ability to perceive and discriminate one’s own and other’s emotions, is a factor of social effectiveness (Salovey and Mayer, 1990; Barrett and Gross, 2001; Mayer et al., 2016). It is also associated with better DA, predicting relational quality and explaining 48% of the variance in DA (Batool and Khalid, 2012).

Multiple studies have demonstrated significant associations (Dworkin et al., 2018; Rusu et al., 2019) and predictive effects of ER on relationship satisfaction (Bloch et al., 2014), highlighting the protective importance of individual ER capacity for relationship quality (Tani et al., 2015). In contrast, DER show significant negative associations with romantic relationship quality and satisfaction (Harrell, 2015; Rick et al., 2017), as well as with intimacy (Constant et al., 2018). Conversely, DER exhibit a positive association with the risk of rupture/divorce (Klein et al., 2016). Specifically, difficulties in acceptance, impulse, awareness, and selection of regulatory strategies reveal negative correlations with the satisfaction dimension of DA (Rick et al., 2017). Furthermore, DER predict fear of emotional involvement, dependency, and control, as well as impact the perception of partner availability, responsiveness, and commitment, making these skills a target of interest for competence training in therapeutic settings (Tani et al., 2015; Clark, 2018, Unpublished).

## Final Reflection

Human beings are social beings, insofar as their humanity is built and fulfilled within relationships with other human beings. Romantic relationships establish a foundation for sharing a life project as a couple, combining “freedom, intimacy, and connection” (Coimbra de Matos, 2017, p. 5). Thus, participating in a romantic relationship is a deeply human act and a desire for most people (Bookwala, 2015), which makes it a topic of interest in psychology (Spanier, 1976). As such, this article intended to analyze variables involved in the functioning of romantic relationships, by expanding the focus beyond the assessment of satisfaction and trying to demonstrate how romantic attachment and the existence of DER are related to each other and to the perceived level of DA.

Research has shown that romantic relationships can be thought of from the perspective of the attachment theory (Hazan and Shaver, 1987). According to this theory, the individual relational predisposition can be seen as the result of a legacy that has been built since the first interactions, evolving with personal development (Bowlby, 1969; Pascuzzo et al., 2013). Attachment will, thus, be a relevant factor in the experience of relationships throughout life, in a cooperative parallel with the development of affective regulation processes (Cassidy, 1994; Viddal et al., 2017). Based on these assumptions, we sought to explore the relationships between the difficulties in these processes, RA patterns, and DA.

In this review, we attempted to organize current knowledge about the articulation between our target variables, starting by investigating the relevance of DA as an indicator of romantic relationship quality and viability (Montesino et al., 2013). Indeed, DA is a parameter that translates the coordination and adaptation work inherent to the evolution of a romantic relationship, as perceived by the person who lives it (Kendrick and Drentea, 2016), combining aspects of relationship satisfaction, partner consensus, and cohesion (Spanier, 1976). Therefore, an analysis of DA is a way to overcome the limitations imposed by more restricted focuses, such as the assessment of relationship satisfaction. The latter, although important for the success of the relationship (Bookwala, 2015), provides an individualistic perspective and ignores aspects of putting aside self-interest in favor of partner union. As such, relationship satisfaction represents a reduced (Fowers et al., 2016) and inadequate focus, given the complexity of relationship functioning (Roberson et al., 2018).

Thus, we tried to demonstrate that looking at romantic relationships through the perspective of their DA is an advantageous option, as it allows us to obtain a more comprehensive picture of their functioning (Anderson et al., 2014; Kendrick and Drentea, 2016; Farero et al., 2019). It also combines aspects of gratification and well-being with dimensions of difficulty, including tensions and anxieties that may interfere in partners’ coordinated articulation (Busby et al., 1995; Hernandez, 2008; Farero et al., 2019). This seems to be an essential point with regard to the study of romantic relationships, since any relationship is a permanent and dynamic exchange between partners, in an interaction that, among other facets, includes an important degree of emotional interdependence (Karan et al., 2019; Sels et al., 2020).

Therefore, it became important to explore the fundamental role of emotions in the functioning of romantic relationships, because not only do emotions emerge to signal needs and motivate individual behaviors (Elliott, 2012), but they also interfere, in a relevant way, in relationship dynamics (Haase, 2014; Rusu et al., 2019). The impact of emotional experience on relationships is not limited to the individual level, as it affects both partners (Gross, 2001, 2015; Butler and Randall, 2013; Bjureberg et al., 2016). This brings into focus the importance of understanding the mechanisms through which ER influences the DA of couples in romantic relationships.

In addition, ER is not merely an individual process, since it is often influenced by other processes, especially in romantic relationships (Thompson and Bolger, 1999; Niven et al., 2012; Butler and Randall, 2013). Thus, it is necessary to develop conceptual models that entwine the dynamic and dyadic nature of emotional responses within conjugal relationships. The capacity to identify emotions and to express them, as well as empathize and manage more defiant emotions, is essential for an adaptive and healthy relationship (Cordova et al., 2005), leading to increased emotional processing in both partners, as well as in the interaction of their emotional systems.

Indeed, research has shown that a more effective ER is associated with higher quality romantic relationships (Batool and Khalid, 2012; Dworkin et al., 2018; Rusu et al., 2019). Conversely, these relationships are negatively impacted by any regulatory difficulty (Harrell, 2015; Tani et al., 2015; Klein et al., 2016; Rick et al., 2017).

We were able to conclude that the set of conscious and unconscious processes that allow people to adjust their emotional experience organize patterns reflecting relevant individual differences in the functioning of romantic relationships (Fraley and Hudson, 2017; Fraley and Roisman, 2019). These processes, by dictating the way emotions are regulated (Barrett and Gross, 2001; Gross, 2001; Peña-Sarrionandia et al., 2015) and their expression is driven (Dworkin et al., 2018), determine the emotional responses that arise within relationships. Furthermore, these processes can be affected by multiple and diverse DER, related to awareness, understanding, and acceptance of emotions, as well as their management (Gratz and Roemer, 2004). These difficulties show a tendency to produce extreme results that can be harmful to the relationship. An example of this would be anxious hyperactivation, in which excessive demand of the partner’s attention motivates intense emotional reactions that tend to produce the opposite effect, driving the partner away (Estévez et al., 2018; Overall, 2019). An antipodal example is emotional deactivation, by avoiding the experience and expression of emotions (Brenning and Braet, 2013; Gillath et al., 2016; Winterheld, 2016; Simpson and Rholes, 2017; Mikulincer and Shaver, 2019).

In any event, these unregulated emotional manifestations suggest responses guided by relational representations in which trust is impaired, regarding not only the romantic partner, but also the person’s own role and their expectations concerning the relationship (Gillath et al., 2016; Fraley, 2019; Mikulincer and Shaver, 2019). This perception is in line with what is known about the parallel evolution of regulatory mechanisms and relational patterns, whose construction evolves in the common context of dependency harbored by the primary relationship (Cassidy, 1994; Cole, 2014; Viddal et al., 2017), reflecting the quality of care received as a space for internalization of trust in relationships (Bowlby, 1969; Pascuzzo et al., 2013). Research has been reinforcing the association between DER patterns and more insecure, anxious, or avoidant attachment patterns (Brenning and Braet, 2013; Malik et al., 2015; Gillath et al., 2016; Winterheld, 2016; Simpson and Rholes, 2017; Umemura et al., 2018; Mikulincer and Shaver, 2019), leading to reduced levels of relational quality (Li and Chan, 2012; Muraru and Turliuc, 2012; Rennebohm et al., 2017; Mark et al., 2018; Siegel et al., 2018; Birnbaum and Reis, 2019; Mikulincer and Shaver, 2019).

Hence, it is possible to conclude that these variables and their interactions are of fundamental importance in the understanding of romantic relationship functioning. However, there is still a large gap regarding the knowledge of the mechanisms that connect them (Martins et al., 2016). We understand that each individual enters a romantic relationship with a set of personal skills, expectations, and limitations that condition them, in a more or less voluntary way. These factors imprint trends in their options and reactions that impact the experience, quality, and viability of the romantic relationship. This reveals the importance of deepening the knowledge on these mechanisms, toward better informed development and planning of psychological interventions. The inseparability and interdependence shown between attachment styles and the regulatory dimension, in their joint evolution and articulation, and the consequences for the adjustment of romantic relationships, propel several issues that deserve further study. Namely, the possibility of bidirectional effects, which make each of these processes a potential predictor of the other, and open space for investigating these mutual influences. Moreover, considering the relative rigidness that characterizes attachment patterns, another point of focus could be the more constant functional aspects (Fraley, 2002), with DER standing out as factors of greater potential in the exploration of strategies with possible therapeutic utility (Clark, 2018, Unpublished).

Regarding the analysis of DER, this review was limited to general aspects and global effects. It did not explore particular characteristics, possible relationships between different DER, and the individual relationships of each DER with the different romantic attachment patterns. A detailed exploration of the literature is also absent, as to the specific influence of each DER on relationship quality, whether on general DA, or on the different dimensions of satisfaction, consensus, and cohesion. In addition, the coordination of factors is considered only from an individual perspective. Thus, the depth of aspects related to the couple’s dyadic relationship is open for further study. In addition, longitudinal studies can help to understand the growth and the variable effects during the relationship.

Despite these limitations, we present a summary of the current knowledge on the articulation of important relational variables, RA, DER, and DA, whose study has not been given due attention. This review sheds light on these issues, identifying a diverse set of new targets for future investigation in a domain so central to human functioning, as are romantic relationships.

## Author Contributions

MF and VS-G contributed to conception and design of the study. LL wrote the first draft of the manuscript. All authors wrote sections of the manuscript and contributed to manuscript revision, read, and approved the submitted version.

## Funding

Financed by national funds of the National Agency for Science and Technology (FCT - Fundação para a Ciência e a Tecnologia, I.P.), under the UID Financing/05749/2020.

## Conflict of Interest

The authors declare that the research was conducted in the absence of any commercial or financial relationships that could be construed as a potential conflict of interest.

## Publisher’s Note

All claims expressed in this article are solely those of the authors and do not necessarily represent those of their affiliated organizations, or those of the publisher, the editors and the reviewers. Any product that may be evaluated in this article, or claim that may be made by its manufacturer, is not guaranteed or endorsed by the publisher.
